# Hypoxia/pseudohypoxia‐mediated activation of hypoxia‐inducible factor‐1α in cancer

**DOI:** 10.1111/cas.13990

**Published:** 2019-03-23

**Authors:** Yoshihiro Hayashi, Asumi Yokota, Hironori Harada, Gang Huang

**Affiliations:** ^1^ Laboratory of Oncology School of Life Sciences Tokyo University of Pharmacy and Life Sciences Tokyo Japan; ^2^ Divisions of Pathology and Experimental Hematology and Cancer Biology Cincinnati Children's Hospital Medical Center Cincinnati OH USA

**Keywords:** HIF1A, hypoxia, oncometabolite, pseudohypoxia, Warburg effect

## Abstract

Since the first identification of hypoxic cells in sections of carcinomas in the 1950s, hypoxia has been known as a central hallmark of cancer cells and their microenvironment. Indeed, hypoxia benefits cancer cells in their growth, survival, and metastasis. The historical discovery of hypoxia‐inducible factor‐1α (HIF1A) in the early 1990s had a great influence on the field as many phenomena in hypoxia could be explained by HIF1A. However, not all regions or types of tumors are necessarily hypoxic. Thus, it is difficult to explain whole cancer pathobiology by hypoxia, especially in the early stage of cancer. Upregulation of glucose metabolism in cancer cells has been well known. Oxygen‐independent glycolysis is activated in cancer cells even in the normoxia condition, which is known as the Warburg effect. Accumulating evidence and recent advances in cancer metabolism research suggest that hypoxia‐independent mechanisms for HIF signaling activation is a hallmark for cancer. There are various mechanisms that generate pseudohypoxic conditions, even in normoxia. Given the importance of HIF1A for cancer pathobiology, the pseudohypoxia concept could shed light on the longstanding mystery of the Warburg effect and accelerate better understanding of the diverse phenomena seen in a variety of cancers.

## 1 INTRODUCTION

In mammalian cells, the major processes of ATP production from glucose are mitochondrial electron transport and oxidative phosphorylation, both of which depend on oxygen. The tricarboxylic acid (TCA) cycle in mitochondria is also an oxygen‐dependent system as some co‐enzymes, such as NAD, are provided from the mitochondrial electron transport system. Glucose metabolism is generally activated in tumor cells. Notably, many tumor cells are known to utilize glycolysis, an oxygen‐independent process, even in the normoxia condition.^1^ This phenomenon, known as the Warburg effect, was first identified by Otto Warburg in the 1920s.^1^

The impact of hypoxia on cancer pathogenesis has also been well documented since the report by Thomlinson and Gray in the 1950s.^2^ They identified the presence of hypoxic cells surrounding the necrotic tumor center in histological sections of carcinomas.^2^ Several decades after this first description of hypoxic cells, Wang and Semenza discovered hypoxia‐inducible factor‐1α (HIF1A), a critical transcription factor for hypoxia adaptation.^3^ Although HIF1A was initially identified as a key factor for response to hypoxia and many phenomena in hypoxic response result from HIF1A signaling activation,^4^, ^5^, ^6^ recent accumulating evidence has revealed a variety of hypoxia‐independent mechanisms for HIF1A signaling activation.^7^, ^8^ These mechanisms could induce a pseudohypoxic condition even when a sufficient level of oxygen is present. The coined term “pseudohypoxia” was originally used for the phenomena of hypoxia‐like metabolic changes in diabetes.^9^ As many phenomena in hypoxia could be explained by HIF1A, hypoxia‐independent activation of HIF1A signaling could mimic many hypoxia‐mediated phenomena, even in the normoxia condition. Thus, these mechanisms underlying oxygen‐independent activation of HIF1A signaling are now termed pseudohypoxia.^10^, ^11^, ^12^, ^13^ From this perspective, we describe an overview of pseudohypoxia in cancer and recent findings.

## 2 OXYGEN‐DEPENDENT ACTIVATION OF HIF1A

**Figure 1:**
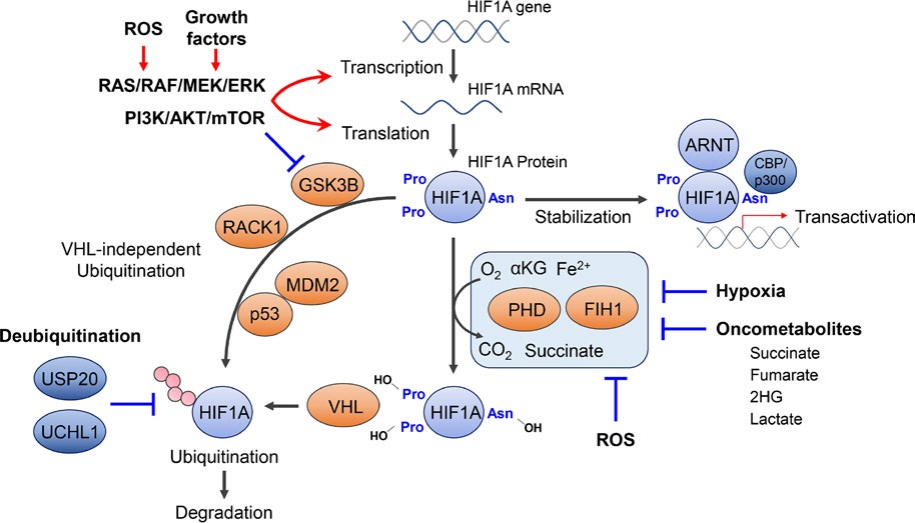
Regulation of hypoxia‐inducible factor 1 α (HIF1A) in hypoxia and pseudohypoxia. Expression of HIF1A is tightly controlled at transcriptional, translational, and posttranslational levels. The stability of HIF1A protein is regulated by the oxygen‐dependent prolyl hydroxylase domain protein (PHD)‐von Hippel‐Lindau tumor suppressor protein (VHL) axis. HIF1A subunit inhibitor FIH1 also represses HIF1A transcriptional activity. These critical enzymes for posttranslational modification of HIF1A require oxygen, Fe^2+^, and α‐ketoglutarate for their catalytic reaction. Thus, as well as oxygen, several oncometabolites can inhibit these enzymes. Mouse double minute 2 homolog (MDM2)/p53, receptor for activated C kinase 1 (RACK1), and glycogen synthase kinase‐3β (GSK3B) are involved in the VHL‐independent ubiquitination processes for HIF1A protein. Deubiquitination of HIF1A protein could also affect HIF1A protein stability. ARNT, aryl hydrocarbon receptor nuclear translocator; Asn, asparagine; CBP, CREB‐binding protein; Pro, proline; ROS, reactive oxygen species; UCHL1, ubiquitin C‐terminal hydrolase‐L1; USP20, ubiquitin specific peptidase 20

Hypoxia‐inducible factor‐1α was originally identified as a critical factor for cellular adaptation to hypoxic conditions. Now it is well known that HIF1A regulates a variety of physiologic pathways, such as hematopoietic stem cell regulation, cell proliferation, survival, apoptosis, angiogenesis, glucose metabolism, and also immune cell activation.^4^, ^5^, ^6^ The number of HIF1A‐regulated genes exceeds 1000 as new cell types and conditions are analyzed by new techniques.^14^ Thus, dysregulation of HIF1A signaling could result in a variety of pathological conditions. We note here that the *EPO* gene is a target of EPAS1 (also known as HIF2A), but not HIF1A, although it was initially considered a HIF1A target.^15^, ^16^ Hypoxia‐inducible factor‐1α is ubiquitously expressed and the expression of HIF1A is tightly controlled at transcriptional, translational, and posttranslational levels (Figure 1).^8^, ^17^ Among these, posttranslational modification is the most critical HIF1A regulation. The stability of HIF1A protein is regulated by the oxygen‐dependent degradation domain through hydroxylation of proline residues 402 and 564 by prolyl hydroxylase domain proteins (PHDs).^18^, ^19^ These modifications favor interaction with the von Hippel‐Lindau tumor suppressor protein (VHL) and subsequent proteasomal degradation.^18^, ^19^ Ubiquitously expressing HIF1A subunit inhibitor HIF1AN (also known as FIH1) could also repress HIF1A transcriptional activity under normoxia by hydroxylating the Asp site 803 of HIF1A protein.^20^, ^21^, ^22^, ^23^ These critical enzymes for posttranslational modification of HIF1A require oxygen for their catalytic reaction. Thus, hypoxia could inhibit those posttranslational modifications of HIF1A, stabilize HIF1A protein, and also keep HIF1A transcriptional activity.

As described below in detail, accumulating evidence has revealed multiple cases of hypoxia‐independent activation of HIF1A signaling (Figure 1).^8^ Indeed, we have recently shown that pseudohypoxia‐mediated HIF1A signaling activation is a central pathobiological mediator of myelodysplastic syndromes (MDS), a group of clonal hematopoietic disorders characterized by ineffective hematopoiesis and multilineage dysplasia.^7^

## 3 OXYGEN‐INDEPENDENT HIF1A PROTEIN STABILIZATION

### 3.1 *VHL* mutations

Loss‐of‐function germline mutations in the *VHL* gene cause von Hippel‐Lindau disease, an inherited disorder characterized by abnormal growth of multiple tumors and cysts in the body.^24^ Hemangioblastoma in the central nervous system, clear cell renal carcinoma, and pheochromocytoma are frequently observed in patients with VHL disease.^24^ Somatic mutations in the *VHL* gene or inactivation of *VHL* gene expression are also common in a majority of patients with sporadic clear cell renal carcinoma.^25^ Given that VHL is a critical E3 ubiquitin ligase, which recognizes PHD‐mediated hydroxylation of proline residues, for oxygen‐dependent HIF1A protein degradation,^18^, ^19^ defective function of VHL causes the stabilization and accumulation of HIF1A protein even in normoxia. Additionally, VHL could function as a repressor of HIF1A transcriptional activity under hypoxia.^20^

### 3.2 MDM2 and TP53 axis

Mouse double minute 2 homolog (MDM2) E3 ubiquitin ligase promotes HIF1A protein degradation regardless of the oxygen condition. MDM2 cooperates with tumor suppressors, such as p53, to downregulate HIF1A protein expression. MDM2 is known to be an E3 ubiquitin ligase of p53 protein.^26^ In normoxia, it was reported that HIF protein could bind to the p53 protein and undergo ubiquitination by MDM2 and proteasomal degradation.^26^ Thus, loss‐of‐function mutation in the *TP53* gene could affect MDM2‐mediated oxygen‐independent regulation of HIF1A degradation, leading to accumulation of HIF1A protein. Recently, we have shown that *RUNX1* mutant could stabilize HIF1A protein by disrupting MDM2/p53 axis in normoxia.^7^ Cai et al^27^ showed decreased p53 protein (but not mRNA) expression levels and ribosome biogenesis in *Runx1*‐deficient hematopoietic stem cells and progenitors. It has been shown by others that defective ribosome biogenesis causes MDM2 inactivation in 5q‐syndrome (a discrete subtype of MDS). This might explain the reason why *RUNX1* mutations and *TP53* mutations are mutually exclusive or negatively co‐mutated in the MDS cohort.^28^

### 3.3 RACK1 and HSP90

Heat shock protein 90 (HSP90) binds to the basic helix‐loop‐helix (bHLH)‐PER‐ARNT‐SIM (PAS) domain of HIF1A protein and regulates HIF1A activation.^29^ Receptor for activated C kinase 1 (RACK1) competes with HSP90 for binding to the bHLH‐PAS domain.^30^ It has been reported that disruption of the interaction between HSP90 and HIF1A protein by HSP90 inhibitor could allow RACK1 to bind HIF1A protein, resulting in the recruitment of the E3 ubiquitin ligase complex and degradation of HIF protein.^30^ In this process, phosphorylation of RACK1 and its dimerization is required for Elongin‐C, a major component of the E3 ubiquitin ligase complex for HIF1A degradation. Interestingly, calcineurin, a serine/threonine phosphatase, promotes dephosphorylation of RACK1 and stabilization of HIF1A protein.^31^ Thus, calcineurin inhibitors (cyclosporine A and FK506), widely used immunosuppressive agents in the clinical field, could induce HIF1A protein degradation.^31^

### 3.4 USP20 and UCHL1

Deubiquitination of HIF1A protein also results in stabilization of HIF1A protein. Ubiquitin specific peptidase 20 (USP20, also known as VDU2) has been reported to specifically deubiquitinate and stabilize HIF1A protein.^32^ Ubiquitin C‐terminal hydrolase‐L1 (UCHL1) is also a well‐studied deubiquitinase and its association with multiple cancers has been reported.^33^ Recently, UCHL1 was identified as a novel regulator of HIF1A signaling activation.^34^ UCHL1 could abrogate VHL‐mediated HIF1A ubiquitination. Notably, the expression level of UCHL1 in breast and lung cancers was positively correlated with that of HIF1A and poor prognosis.^34^

## 4 TRANSCRIPTIONAL AND TRANSLATIONAL REGULATION OF HIF1A

Although HIF1A is mainly regulated at the protein level, HIF1A is also regulated at the transcriptional and translational levels. Activation of the PI3K/AKT/mTOR signaling cascade is well known to upregulate both HIF1A mRNA transcription and HIF1A protein translation.^14^, ^35^ This means that multiple growth factors, activation of oncogenes, and mutations in tumor suppressor genes (such as *PTEN*) could activate HIF1A signaling through the PI3K/AKT/mTOR pathway.^35^ Glycogen synthase kinase‐3β (GSK3B) is known to phosphorylate HIF1A protein, leading to degradation of HIF1A protein in a VHL‐independent manner.^17^ Notably, PI3K/AKT signal could inactivate GSK3B, resulting in HIF1A protein stabilization.^17^ Interestingly, GSK3B deficiency in hematopoietic stem cells has been reported to cause MDS.^36^ Considering our recent report showing HIF1A is a critical mediator of MDS pathogenesis,^7^ GSK3B deficiency could cause MDS phenotypes through HIF1A signaling activation.

Several growth factors and oncogenic events could also activate RAS signaling through the RAS/RAF/MEK/ERK cascade, leading to acceleration of HIF1A protein synthesis.^8^ Notably, ERK activation also enhances HIF1A transcriptional activity through phosphorylation of CBP/p300, a critical co‐factor for HIF1A transcriptional activity.^8^

## 5 REACTIVE OXYGEN SPECIES‐MEDIATED HIF1A SIGNALING ACTIVATION

Reactive oxygen species (ROS) are involved in HIF1A signaling activation. In the late 1990s, it was shown that mitochondria generates abundant ROS under hypoxic conditions and this could activate HIF1A signaling.^37^ This finding was further confirmed by several studies,^38^, ^39^ in which PHD inactivation was proposed as a mechanism of the ROS‐mediated HIF1A signaling activation. However, the mechanisms of ROS‐mediated stabilization of HIF1A in hypoxia remain to be elucidated.^40^, ^41^ In contrast, it is evident that ROS could stabilize HIF1A protein under normoxia.^39^, ^40^ Oxidization of Fe^2+^, a co‐factor of PHDs, by ROS^42^ has been proposed as a mechanism of this. Reactive oxygen species could also upregulate HIF1A transcription and translation through ERK and PI3K/AKT/mTOR signaling pathways or induction of microRNA‐210.^40^

## 6 ONCOMETABOLITES FOR HIF1A SIGNALING ACTIVATION

### 6.1 Succinate and fumarate

Besides oxygen as a substrate, both PHDs and FIH1 require α‐ketoglutarate (α‐KG) as a co‐factor for their enzymatic reaction.^43^ Thus, PHDs and FIH1 are called α‐KG‐dependent dioxygenases. As α‐KG is an intermediate metabolite of the mitochondrial TCA cycle, a decrease in the concentration of α‐KG or accumulation of subsequent metabolites following α‐KG, such as succinate, fumarate, and malate, could affect the activity of α‐KG‐dependent dioxygenases. In the early 2000s, loss‐of‐function mutations in genes encoding for succinate dehydrogenase (SDH) subunits and fumarate hydratase (FH) were identified in patients with several cancers such as pheochromocytoma, paraganglioma, and renal cell carcinoma.^44^, ^45^, ^46^, ^47^, ^48^ Downregulation of SDH has also been reported in several cancers including gastric and colon carcinoma.^49^ Activation of HIF1A was also reported in tumors with *SDHD* mutations.^47^, ^48^, ^50^ In 2005, the important evidence regarding the mechanisms of HIF1A activation by dysregulated SDH and FH was reported from independent groups.^51^, ^52^ These reports actually pioneered a concept of oncometabolites. The dysfunctional SDH and FH result in accumulation of succinate and fumarate. These metabolites competitively inhibit the α‐KG‐dependent PHD catalytic reaction. FIH1 could also be inhibited in this circumstance.

### 6.2 2‐Hydroxyglutarate

Isocitrate dehydrogenases (IDHs) are enzymes that convert isocitrate to α‐KG. In mammalian cells, IDHs consist of 3 isoforms. Among them, IDH1 locates in cytoplasm, whereas IDH2 locates in mitochondria. Both of them catalyze reversible conversion of isocitrate to α‐KG by using NADP^+^ as a co‐enzyme. In 2008 and 2009, mutations in genes encoding IDH1 and IDH2 were identified in the patients with glioblastoma and acute myeloid leukemia.^53^, ^54^, ^55^ To date, IDH mutations have been reported in a variety of cancers, such as angioimmunoblastic T‐cell lymphoma, myelodysplastic syndromes, myeloproliferative neoplasms, cholangiocarcinoma, and chondrosarcoma.^56^, ^57^ Mutant IDHs lose their initial catalytic function for the conversion of isocitrate to α‐KG, and gain the function for the production of 2‐hydroxyglutarate (2HG) from α‐KG and NADPH.^58^, ^59^ 2‐Hydroxyglutarate is a normal byproduct of mitochondrial metabolism.^58^, ^59^ Although its physiological role remains unknown, the concentration of 2HG in normal cells is maintained at very low levels.^59^ There are 2 enantiomers of 2HG, D(*R*)‐2HG and L(*S*)‐2HG.^58^, ^59^ Mutant IDHs cause an aberrant accumulation of D(*R*)‐2HG.^60^, ^61^ On the other hand, it has recently been shown that L(*S*)‐2HG can be produced in response to hypoxia.^62^ Importantly, both of the 2HG enantiomers could competitively inhibit α‐KG‐dependent dioxygenases due to the structural analogy between 2HG and α‐KG.^63^ However, it has also been reported that D(*R*)‐2HG, but not L(*S*)‐2HG, rather stimulates PHD activity resulting in degradation of HIF1A protein in astrocytes.^64^ It might depend on cell types, conditions, or other factors. Thus, there is still controversy regarding the effect of 2HG enantiomers, especially D(*R*)‐2HG, on PHDs and HIF1A regulation.

### 6.3 Lactate

Hypoxia‐inducible factor‐1α is known to be a critical regulator of glycolysis as multiple key enzymes of the glycolysis pathway are direct targets of HIF1A. Lactate dehydrogenase (LDH) is a tetramer which is constituted by 2 subunits, LDHA and LDHB.^65^ Both LDHA and LDHB are induced by HIF1A, and involved in the conversion between pyruvate and lactate, a glycolytic end product. Notably, LDHA primarily converts pyruvate to lactate, whereas LDHB converts lactate to pyruvate.^65^ It has been reported that lactate is involved in cell migration, invasion, immune escape, and radioresistance of cancer cells.^66^ Recently, it was reported that circulating lactate is converted to pyruvate by LDHB and could be the primary carbon source for the mitochondrial TCA cycle.^67^, ^68^ This indicates that the glycolytic end product can be effectively used as a fuel for cancers, resulting in the growth advantage of cancer cells.^67^ Importantly, lactate could induce HIF1A activation thorough inhibition of PHD‐mediated proline hydroxylation.^69^, ^70^ Pyruvate has also been reported to induce pseudohypoxic conditions.^71^

## 7 HYPOXIA AND PSEUDOHYPOXIA IN CANCER

**Figure 2:**
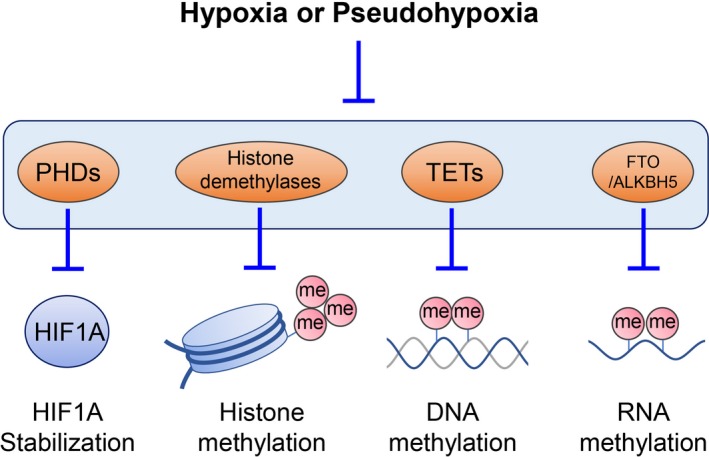
Hypoxia‐inducible factor‐1α (HIF1A) signaling activation and epigenome hypermethylation in hypoxia and pseudohypoxia. Hypoxia and pseudohypoxia (especially oncometabolites, such as succinate, fumarate, and 2‐hydroxyglutarate could inhibit the prolyl hydroxylase domain (PHD)‐von Hippel‐Lindau tumor suppressor protein axis and HIF1A subunit inhibitor FIH, leading to activation of HIF1A signaling. Hypoxia and pseudohypoxia also inhibit the activity of histone demethylases, tet methylcytosine dioxygenases (TETs), and fat‐mass and obesity‐associated protein (FTO)‐AlkB homolog 5 (ALKBH5) as they are α‐ketoglutarate‐dependent enzymes. me, methyl group

In typical solid tumors, despite the fact that a certain regions of tumors are far removed from proper blood flow because of uncontrolled tumor growth and disorganized vascular formation, hypoxia appears to result in benefiting the tumor growth.^72^, ^73^, ^74^ This indicates that cancer cells might adapt to the stressed condition, overcome it, and gain an advantage for survival and growth. In 1956, Thomlinson and Gray reported the presence of hypoxic cells surrounding the necrotic tumor center in histological sections of solid malignant tumors.^2^ Hypoxia was interpreted as a critical component of the tumor microenvironment.^2^ This report then opened the door to a vast number of subsequent studies related to hypoxia, resulting in an acceleration of uncovering the role of hypoxia in cancer pathogenesis. Hypoxia promotes a physiological selection for the cells with defects in apoptosis, such as the cells acquiring *TP53* mutations.^75^ Hypoxia induces several well‐known hallmarks of cancer, such as aberrant angiogenesis, invasion, metastasis, and epithelial‐mesenchymal transition.^41^ Cellular glucose metabolism,^76^ mitochondrial function,^77^ and production of ROS^38^ are also affected by hypoxic conditions. Acute and chronic hypoxia affect the pathobiology of cancers differently.^78^, ^79^ Furthermore, hypoxia affects the epigenetic networks in tumor cells.^80^, ^81^ The local hypermethylation of the CpG‐rich promotors of the tumor suppressor genes is well known in a wide variety of neoplasms.^82^, ^83^ The tet methylcytosine dioxygenases (TETs) are critical enzymes involved in DNA demethylation through oxidizing 5‐methylcytosine on DNA.^35^ Thus, inactivation of TETs results in DNA hypermethylation. Recently, Thienpont et al^84^ showed that hypoxia causes the reduction of TET activity independently of HIF1A or other hypoxia‐associated alterations. The activity of several histone demethylases of the Jumonji domain‐containing family could also be influenced by hypoxia.^80^ Interestingly, whereas those histone demethylases require oxygen for their catalytic reaction, it has been reported that some of them are induced by hypoxia.^81^ Therefore, there is a controversy on hypoxia‐mediated regulation of histone demethylases. It might depend on the oxygen level, exposure duration to hypoxia, or cell type.^81^, ^85^ Recently, it has been uncovered that not only DNA and histone but also RNA modification is critical for regulation of many physiologic pathways and disease development.^86^ N^6^‐Methyladenosine (m^6^A) is an abundant internal modification in mRNA.^86^ Fat‐mass and obesity‐associated protein (FTO) and AlkB homolog 5 (ALKBH5) are the critical enzymes for m^6^A demethylation.^86^ Notably, FTO and ALKBH5 require oxygen, Fe^2+^, and α‐KG for their enzymatic reaction, suggesting that oxygen status could affect the RNA modification. In addition to the hypoxia condition, oncometabolites could affect several factors for epigenetic regulation (Figure 2). The TETs, FTO, ALKBH5, and histone demethylases of the Jumonji domain–containing family are also α‐KG‐dependent enzymes, and their catalytic activity is inhibited by several oncometabolites described above, such as succinate, fumarate, and 2HG.

## 8 PSEUDOHYPOXIA AND THE WARBURG EFFECT

In mammalian cells, glucose is a major source of energy. Many, if not all, cancer cells utilize glucose more extensively than other normal cells. Indeed, this characteristic feature is clinically applied for the diagnosis of many cancers using 18 F‐fluoro‐2‐deoxy‐d‐glucose PET. In normal cells under physiologic conditions, ATP production from glucose is primarily through oxygen‐dependent mitochondrial electron transport and oxidative phosphorylation rather than oxygen‐independent glycolysis. However, the glycolysis pathway is known to be activated in a wide variety of cancer cells, even in normoxic conditions. This phenomenon (known as the “Warburg effect”) was identified by Otto Warburg in the 1920s and reported later.^1^ The reason why cancer cells prefer to utilize inefficient glycolysis for glucose metabolism under normoxic conditions has been a longstanding mystery. Initially, it was considered that the mitochondrial dysfunction could cause a metabolic shift from oxidative phosphorylation to glycolysis in cancer cells.^1^ This concept actually appears to be widely known. However, as Weinhouse pointed out, and even Warburg himself mentioned later,^87^ the mitochondrial biogenesis of cancer cells is not necessarily defective. It might be actually activated in some cancer cells. Indeed, Hensley et al^67^ recently reported that glucose consumption is activated in lung cancers and both glycolysis and the TCA cycle are activated even in the less perfused regions. This indicates that activation of glucose metabolism is not always synonymous with metabolic switch from oxidative to glycolytic metabolism.

The identification of hypoxic cells in tumors by Thomlinson and Gray and the discovery of HIF1A by Semenza has have provided a certain clue as to the mystery of activated glucose metabolism in cancer cells.^2^, ^3^ Hypoxic conditions could induce HIF1A, a master regulator of glucose metabolism, and activate the expression of key enzymes for glycolysis. It appears to be reasonable to explain the Warburg effect as a part of the adaptation process of cancer cells to hypoxic conditions. However, glucose metabolism is also activated in many cancer cells under normoxic conditions. Now it has become clear that HIF1A signaling can also be activated through hypoxia‐independent multiple mechanisms (Figure 1).^8^ This indicates that glucose metabolism could be activated even in normoxia. Thus, pseudohypoxia‐mediated HIF1A signaling activation might well explain the Warburg effect.

## 9 CONCLUDING REMARKS

Accumulating evidence has revealed a variety of hypoxia‐independent mechanisms for HIF1A signaling activation.^7^, ^8^ As a result of HIF1A signaling activation, the glycolysis pathway is activated, leading to accumulation of pyruvate and lactate. As described above, accumulation of lactate could stabilize HIF1A and also benefit cancer cells by being involved in cell migration, invasion, immune escape, and radioresistance of cancer cells.^41^ Both lactate and pyruvate could be substrates for the mitochondrial TCA cycle in cancer cells.^67^, ^68^, ^88^ In addition to genes related to the glycolysis pathway, HIF1A regulates many critical genes involved in tumor progression, metastasis, and resistance to therapies. Thus, activation of HIF signaling, regardless of the oxygen situation, is advantageous to cancer cells for their survival and development.

## CONFLICT OF INTEREST

The authors declare no conflict of interest.
